# Affinity and Matrix Effects in Measuring Fish Plasma Vitellogenin Using Immunosorbent Assays: Considerations for Aquatic Toxicologists

**DOI:** 10.5402/2012/942804

**Published:** 2012-09-18

**Authors:** Stephen E. Bartell, Heiko L. Schoenfuss

**Affiliations:** ^1^Aquatic Toxicology Laboratory, Saint Cloud State University, WSB-273, 270 Fourth Avenue South, St. Cloud, MN 56301, USA; ^2^Department of Biology, Normandale Community College, Bloomington, MN 55431, USA

## Abstract

Enzyme-linked immunosorbent assays (ELISAs) are important tools in aquatic toxicology and have become crucial in assessing exposure concentrations in the aquatic environment and acute physiological responses in exposed organisms. These assays utilize the inherent properties of antibodies to recognize and selectively bind a target molecule, while largely ignoring other molecules to provide semiquantitative values. A variety of methodologies to measure plasma vitellogenin using ELISAs have generated widely divergent data. Limitations of the ELISA method are known in the wider immunology field, though aquatic toxicologists may be less familiar with these limitations. We evaluated several mechanisms contributing to the divergent vitellogenin data in the literature. Antibody affinities and the matrix in which standard curves are constructed are possible error generators. These errors can be amplified by large sample dilutions necessary to fall within the standard curve. It is important for the aquatic toxicology research community to realize the limitations and understand the pitfalls of absolute plasma vitellogenin data in their studies.

## 1. Introduction

Enzyme-linked immunosorbent assays (ELISAs) are important tools in aquatic toxicology as their low detection limits allow for the fast and relatively inexpensive measurements of many compounds in complex environmental matrices (water, effluent) and biological samples (blood, urine, homogenates). As aquatic toxicologists struggled with the subtle effects of ng/L concentrations of contaminants of emerging concerns, ELISAs became a central tool in assessing exposure concentrations in the aquatic environment and acute physiological responses in exposed organisms. These assays utilize the inherent properties of antibodies to recognize and selectively bind a target molecule, while largely ignoring other molecules to provide semiquantitative values. Commercially available assays have been developed to measure, for example, 17 *β*-estradiol at ng/L concentrations in water or 4-nonylphenol and carbamazepine at higher ng/L and *μ*g/L concentrations. A plethora of ELISAs are commercially available for vertebrate physiological endpoints such as serum cortisol concentrations and other steroid hormone concentrations. 

One target of particular prominence to the study of endocrine active compounds is the egg-yolk precursor protein vitellogenin, which is naturally produced in mature female oviparous vertebrates and may be produced in males in the presence of exogenous estrogens and then serves as an indicator of acute exposure [1]. Although the ability of vitellogenin induction in male fishes to predict adverse health effects of exogenous estrogen exposure is unresolved ([2, 3]; however, see [4] for predictive modeling), the analysis of plasma vitellogenin in studies of endocrine disruption has become commonplace. Furthermore, vitellogenin has been suggested as a regulatory endpoint with important environmental and financial consequences if approved for such purposes.

**Figure 1:**
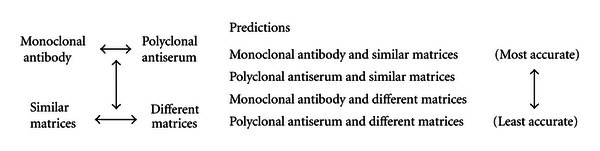
Concept map of interactions between antibody specificity and matrix composition in determining likely accuracy of calculated vitellogenin concentrations for a given plasma sample. Predictions of accuracy of an ELISA using a specific antibody and similar or different matrices follow the concepts of antibody affinity and likelihood that plasma proteins will interfere with the recognition of the target molecule by the antibody.

A review of the published literature reporting vitellogenin concentrations for fishes exposed in laboratories or collected during field studies reveals widely diverging absolute concentrations, sometimes varying by thousand-fold factors [5–9]. Differences in reported values are not explainable solely by intraspecies variability, as a result, for example, of differing degrees of maturity and reproductive status [1, 2], but rather suggest assay-related methodological differences. Several different methodologies have been used to measure vitellogenin values in recent years. Direct ELISA [10, 11] and competitive ELISA using either fathead minnow antibody [9, 12, 13], or crossreactive carp antibody [3, 7, 14], have generated a wide range of plasma vitellogenin concentrations in similarly exposed fathead minnows. However, as ELISA-derived vitellogenin data are becoming more widely applied to decision-making processes, it is important to explore possible methodological mechanisms that may result in divergent relative and absolute vitellogenin measurements. Limitations of immunoassay methods are not restricted to the field of aquatic toxicology, as immunologists in diabetes research have struggled for years with immunoassay standardizations [15–19]. From the perspective of an assay development team, discussions center on linearity, recovery, accuracy, and crossreactivity [15, 18]. Discussions regarding the proper vitellogenin assay have appeared in the literature in recent years, owing to the large variations in reported vitellogenin concentrations [20–23]. Identical laboratory protocols produce vitellogenin data in interlaboratory comparisons with coefficients of variation approaching 40% [24, 25]. However, to scientists studying endocrine disruption in oviparous vertebrates needing to include vitellogenin screening in their study, an understanding of the interpretive power of the data is paramount. Much has been written regarding the need for a centralized vitellogenin assay [12, 26]. A centralized assay for a single species is a remote possibility; however, the number of fish species utilized in endocrine disruption research complicates the single assay notion [26]. A need exists, therefore, to illuminate some of the interassay differences and provide aquatic toxicologists with information to assist in interpreting published data generated with ELISA-based assays (Figure 1).

Several methodological mechanisms exist that could alter the measured concentration from the absolute concentration of vitellogenin in an organism. Among those, differences in binding affinity between polyclonal antiserum and monoclonal antibody are well recognized among immunologists [27]. A polyclonal antiserum is a preparation of antibody molecules with varying specific recognition targets (epitopes) while a monoclonal antibody is a preparation of antibody molecules all possessing the identical specific epitope. Nearly all antigenic proteins, vitellogenin included, will have multiple epitopes. Recognition by these two antibody preparations will produce divergent results (Figure 1).

In addition to antibody preparation, the matrix in which vitellogenin is assessed may affect final measured concentrations. In most studies, a standard curve is established with purified vitellogenin in a buffer solution while vitellogenin from study specimens is usually measured in blood plasma or a whole organism homogenate. When combined, these two mechanisms may account for much of the absolute intraspecies variability encountered in published studies using vitellogenin induction in male fish as a bioindicator of acute exposure to emerging concern.

Consequently, in this study we tested the hypothesis that measured concentrations of vitellogenin will differ between ELISAs using polyclonal antisera versus monoclonal antibodies for identical samples. In a competitive ELISA, the concentrations garnered with polyclonal antisera were predicted to be higher relative to those values obtained with monoclonal antibody usage.

Consideration of the matrix effect generates a second hypothesis. The addition of plasma from unexposed fish to the standards will result in a downward shift of the standard curve, resulting in lower calculated vitellogenin concentrations when compared to a protocol employing the normal method of preparing a standard curve in a buffer.

## 2. Materials and Methods

### 2.1. Organisms

Plasma was obtained from three unexposed fathead minnows and used to evaluate each methodology. Six-month-old mature male fathead minnows (*Pimephales promelas*) were obtained from a laboratory fish supplier (Environmental Consulting and Testing, Superior, WI) and maintained following established US EPA guidelines [28] (16 : 8 h light:dark, 21°C water temperature). Fish were fed frozen brine shrimp (*Artemia franciscana, *San Francisco Bay Brand, Inc., Newark, CA) twice daily *ad libitum*. Animal care and use in all experiments was approved by the St. Cloud State University Animal Care and Use Committee (IACUC).

To collect whole blood, fish (*n* = 3) were deeply anaesthetized in 0.1% MS-222 and fish tails were severed to harvest blood (approximately 140 *μ*L/fish) using heparinized microhematocrit tubes. Blood was immediately centrifuged to isolate plasma (5,000 g for 5 min at 20°C-same temperature as the harvested fish blood), and the plasma was placed on ice and analyzed by ELISA for vitellogenin. Plasma samples from the three fish were maintained and analyzed separately for the three fish throughout the study providing three independent replications.

### 2.2. Experiment 1-Polyclonal Antiserum versus Monoclonal Antibody

Two ELISAs were used in the experiments. Both were competitive antibody-capture assays using either a polyclonal antiserum preparation or a monoclonal antibody. The polyclonal antiserum was raised against purified fathead minnow vitellogenin [29] and used at a dilution of 1 : 10000. The monoclonal antibody was purchased from Biosense (Bergen, Norway) and used at a dilution of 1 : 1250. The assay consists of microtiter wells coated with purified fathead minnow vitellogenin at a concentration of 600 ng/mL in carbonate coating buffer (pH 9.6) for several hours at room temperature. A preincubation step combines the antibody 1 : 1 with either purified fathead minnow vitellogenin standard or sample plasma diluted in PBS/1% BSA for 1 hour at 20°C. Final dilutions of antibody were 1 : 20000 (polyclonal) or 1 : 2500 (monoclonal). Prior to adding this preincubation mixture to the wells, the plates were washed three times with PBS-Tween using an automated plate washer. Two hundred microliters of the preincubation mixture was added to the vitellogenin coated-wells and incubated at room temperature for 1 hour. Wells were washed with PBS-Tween three times, and detection was via horseradish-peroxidase- (HRP-) labeled secondary antibody, either anti-rabbit IgG-HRP in the polyclonal version or anti-mouse IgG-HRP in the monoclonal version, at 1 : 10000 for I hour at RT. The substrate was tetramethylbenzidine (TMB, Sigma, St. Louis, MO), and plates were read via a Multiskan EX (Thermo Fisher, Waltham, MA) plate reader at 630 nM. Data were analyzed using Ascent Software for Multiskan, version 2.6. A four-parameter logistic regression was used to construct seven-point standard curves.

Split plasma samples from each of the three fish were used in both types of ELISA. The samples were tested by ELISA to verify that vitellogenin concentrations were below the lower detection limit of the assay (detection range: 3.75 *μ*g/mL to 4.8 mg/mL). Each plasma sample was spiked with 300 *μ*g/mL purified vitellogenin and tested to ascertain detection by the two ELISA methods. Pooled estradiol-exposed male fathead minnow plasma (following a 21-day flow-through exposure to 50 ng/L 17 *β*-estradiol) was included in both ELISA methods as a positive control.

### 2.3. Experiment 2-Matrix Effects

The objective of the second experiment was to test the effect of standard curve preparation and included the same split, spiked plasma samples from the first experiment (Section 2.2). Standard curves were generated using three separate protocols: the standard procedure of diluting standards in PBS/1% BSA, and two variations of fathead minnow plasma replacing the PBS/BSA buffer (plasma substitution protocols). The normal procedure of standard preparation consists of standard vitellogenin diluted in PBS/1% BSA dilution buffer. Vitellogenin stock standard (600 *μ*g/mL) was diluted 1 : 62.5 in this dilution buffer to create the working stock with a concentration of 9.6 *μ*g/mL. This working stock was then serially diluted in a seven step two-fold manner, creating a range of standards from 4.8 *μ*g/mL to 0.075 *μ*g/mL vitellogenin. The diluted plasma substitution protocol consisted of pooled plasma from numerous unexposed fish added to PBS/1% BSA at a 1 : 8 dilution. The pooled plasma was tested by ELISA to verify that vitellogenin concentrations were below the lower detection limit of the assay (detection range: 1.95 *μ*g/mL to 2.4 mg/mL). The vitellogenin stock standard (600 *μ*g/mL) was added to this 1 : 8 plasma/PBS at a 1 : 62.5 dilution to create a working stock, and this working stock was then serially diluted 1 : 1 for 7 steps in PBS/1% BSA. The standard range was 4.8 *μ*g/mL → 0.075 *μ*g/mL vitellogenin, while the effective plasma dilution was 1 : 16 → 1 : 1024. This plasma dilution range was selected to approximate the dilution of plasma in tested samples, which were diluted 1 : 50, 1 : 250 and 1 : 1000. The constant plasma substitution protocol consisted of the prepared standards from the normal method added to equal volumes of 1 : 250 pooled plasma. The effective dilution of plasma was 1 : 500, again selected to approximate the dilution of plasma in tested samples. The range of this set of standards was 2.4 *μ*g/mL → 0.075 *μ*g/mL.

All three standard curve methods utilized separate maximum binding, blank and BSA coated well controls, prepared according to the standard curve method. The BSA-coated wells were coated with 1% BSA instead of purified vitellogenin to demonstrate lack of BSA recognition by the antibodies. Each standard was prepared and then split to test in the polyclonal and monoclonal ELISA variation. Given the objective of the study, the number of replicates (*n* = 3) and the range of calculated concentrations (Table 1) only qualitative comparisons were performed.

## 3. Results

### 3.1. Experiment 1-Polyclonal Antiserum versus Monoclonal Antibody

**Figure 2:**
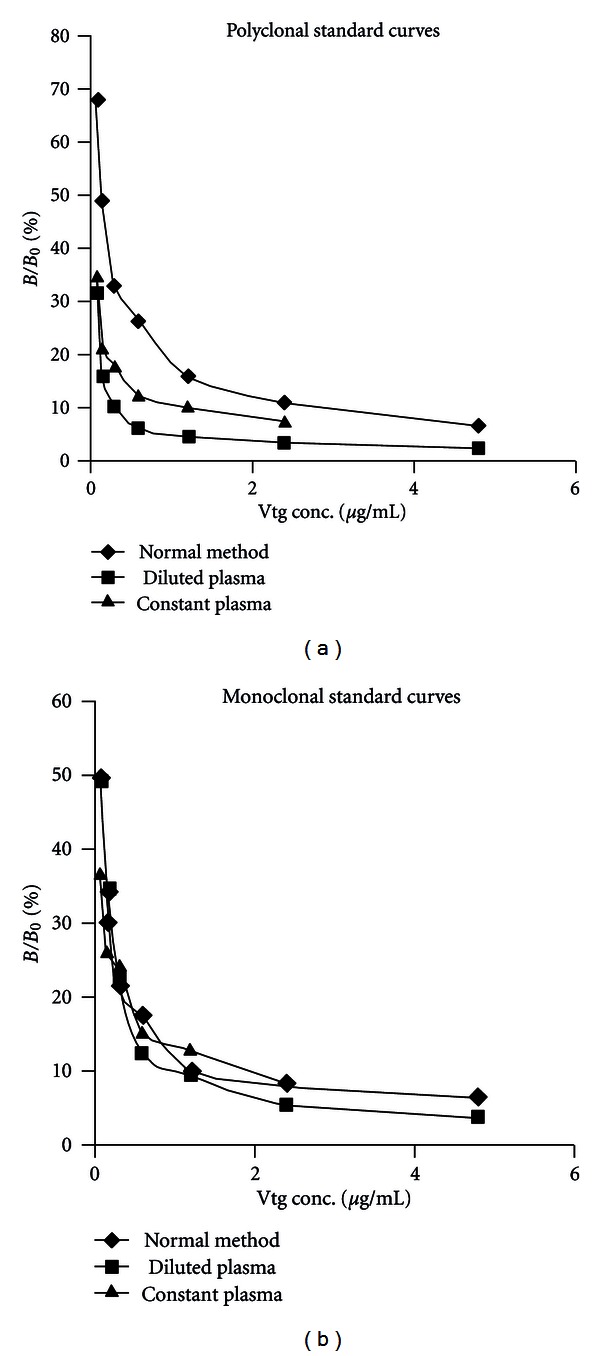
Standard curves generated using polyclonal antisera (a) or monoclonal (b) antibodies against fathead minnow vitellogenin. Standard vitellogenin was diluted across seven points in three different matrices. Normal method: prepared in phosphate-based dilution buffer. Diluted plasma: unexposed plasma substituted for dilution buffer, where the plasma was diluted with the standards across a range of 1 : 16 to 1 : 1024. Constant plasma: unexposed plasma substituted for dilution buffer with standards added, where the plasma was diluted 1 : 500. Unexposed fathead minnow plasma confirmed by ELISA for undetectable vitellogenin at a detection limit of 3 *μ*g/mL.

Standards were prepared in phosphate dilution buffer and split into equal fractions, then used to prepare standard curves in competitive ELISAs using either a polyclonal or monoclonal antibody for binding (Figure 2). Regression analysis of the standard curves was used to calculate vitellogenin values of the spiked plasma samples. The polyclonal antiserum produced calculated amounts 156% higher on average than those calculated with the monoclonal antibody (Table 1).

### 3.2. Experiment 2-Matrix Effects

To test the effect that plasma may have on the ability of the assay to accurately ascertain vitellogenin concentrations in fish plasma samples, plasma from unexposed fish was added in place of the usual PBS-based assay buffer. Both plasma substitution schemes resulted in visible shifts of the standard curve compared to the standards prepared in the usual manner with PBS-based assay buffer (Figure 2). The effect was more pronounced when a polyclonal antiserum was used. Effects that a shift of the standard curve can have are illustrated in the calculated values of vitellogenin in the spiked plasma samples (Table 1). Regardless of the selection of antibody (polyclonal or monoclonal), the calculated vitellogenin concentrations were less when calculated using a plasma substituted standard curve (Table 1).

## 4. Discussion

ELISAs are an important tool to determine concentrations of compounds in environmental and biological samples at very low (*μ*g/L or ng/L) concentrations. The number of compounds for which antibodies have been developed is substantial, antibody development is ongoing, and custom-antibody production through service laboratories has become inexpensive. ELISA kits for many compounds of interest to aquatic toxicologists have been developed and have been optimized to allow even novices with minimal training and equipment to use ELISAs in their research. As a result, aquatic toxicologists in academia, government agencies, and industry routinely turn to ELISAs to assess the presence and effects of endocrine active compounds in the environment. However, many users of immunoassay techniques are not familiar enough with the pitfalls and limitations of this technique and may misinterpret the resultant data sets. Clinical studies on diabetes involving insulin and insulin antibody measurements have been hampered for over twenty years due to discordance in results generated by different laboratories [19, 30]. Attempts to remedy the disparities have not been overly successful and have included interlaboratory comparisons of identical plasma samples [15], using polyclonal antisera versus monoclonal antibodies [16], using identical assay kits in different labs [15], and use of a common reference standard among labs [15, 18]. Through two experiments, we tested the hypotheses that (i) polyclonal antisera overestimate vitellogenin concentrations in fish plasma and that (ii) matrix effects will further diverge measured vitellogenin concentrations from actual blood plasma concentrations. 

### 4.1. Polyclonal Antiserum versus Monoclonal Antibody

Ideally an assay should consist of the antibody binding solely to the target molecule. A polyclonal antiserum is a preparation in which a test animal such as a rabbit or goat is injected with the immunogen, and the resulting test bleeds contain several to numerous reactive antibody molecules [31]. The injected immunogen in most cases is prepared from fish exposed to a chemical capable of producing the molecule of interest, such as estradiol used to stimulate vitellogenin production [32–34]. Any substance contained in the injection capable of eliciting the immunogenic response will have an antibody produced against that substance. The multiple antibody molecules, of different specificities, will all be contained within the preparation. As a result, the polyclonal antiserum will also contain antibodies specific for nontarget molecules, such as plasma proteins not removed during the immunogen purification process. The vitellogenin protein of at least two oviparous vertebrates has been immunologically separated into differentially reactive polypeptides [35, 36]. Considering the biological impact of estradiol, there are likely other proteins upregulated by estrogens [37], potentially resulting in antibodies not specific to vitellogenin in the polyclonal preparation. These antibodies then have the potential to disrupt the accurate quantification of vitellogenin (Figure 1).

Monoclonal antibodies seem to be the more accurate target detector, though considerations exist for this avenue as well. In the production of monoclonal antibodies, the same initial step of immunizing a test animal, usually a mouse, with plasma of exposed fish will generate numerous antibodies of varying specificities, that is, the polyclonal response. Harvesting antibodies from the immunized test animal involves the removal and homogenization of the spleen. This homogenate is then mixed with cell-cultured lymphocytes to create immortal hybrid cell cultures from which antibodies can be harvested in perpetuity. The rather crude process of the fusion of millions of spleen cells and millions of cultured cells will yield hybrids at random. Through the nature of the vertebrate immune system, any single lymphocyte will produce antibodies of only a single specificity, effectively ignoring the multitude of plasma proteins present in the immunogen. It is essential to isolate a single hybrid cell via dilution techniques to guarantee the antibodies produced are of a single specificity. This single cell is then allowed to multiply to large numbers, ensuring a long-term supply of the desired antibody. Performed correctly, production of a monoclonal antibody will result in antibody molecules of a single specificity. If the hybrid cell is not truly isolated before the growth phase, other antibody specificities will be present in the preparation. As with polyclonal preparations, the existence of additional, possibly contaminating antibodies will alter the accuracy of an assay utilizing a monoclonal antibody. This will be amplified, as in the case of polyclonal usage, when the standard curve is produced using purified vitellogenin in a dilution buffer. 

### 4.2. Matrix Effects

The matrix in which the standard curve is established can also alter measured vitellogenin concentrations in any equilibrium assay (Figure 1). Plotting known concentrations of purified vitellogenin easily yields a robust standard curve with very high r-squared values. In nearly every case, this standard curve is prepared with purified vitellogenin in a phosphate dilution buffer. This medium is very different from that of fish plasma. Other proteins in fish plasma have the potential to disrupt the recognition and binding of the target molecule by the antibody, either by nonspecifically binding to the antibody or by simple steric hindrance. ELISA methods incorporating sufficient incubation times alleviate this concern [38]. Of potentially greater effect are plasma proteins for which there exist reactive antibodies in a polyclonal antiserum preparation.

A potential amplifier of this effect is that, in normal assay protocol, the standards are prepared in some type of dilution buffer such as PBS with BSA. The purified standards are diluted in this buffer to constitute the range for the standard curve. Then the samples are diluted to appropriate dilution factors and assayed to compare against the standard curve and determine a concentration. However, the animal plasma will contain numerous other proteins with the potential to be recognized by an antibody and bound, thereby factoring into the final concentration determination. This problem is addressed in ELISAs such as those used to determine 17 *β*-estradiol concentrations by the suggestion that the samples are purified prior to assaying to remove any contaminating aromatic compounds if it appears that there is contamination present [39, 40]. Applying this to fish plasma in the determination of vitellogenin is not practical, owing to the inability to separate vitellogenin from the remaining proteins, while having the original concentrations upheld.

To determine if contaminating entities are present in vitellogenin assays, the second experiment evaluated the effect of plasma being added to the standard curve preparations. This addresses the validity of calculated concentrations of vitellogenin from fish plasma samples when calculated from a standard curve of purified vitellogenin in phosphate buffer. In testing the effect that a different matrix has on the reliability of a standard curve, plasma was spiked with purified vitellogenin. Previous analyses in this ELISA showed good recovery (77–117%) when purified vitellogenin spikes were prepared in a PBS dilution buffer (Table 2). In this study, unexposed plasma samples were spiked with 300 *μ*g/mL purified vitellogenin. Regression analysis using a standard curve constructed in PBS dilution buffer and using a polyclonal antiserum produced calculated values over 600% higher on average than the spiked amount (Table 1). Using a monoclonal antibody, the calculated value was 180% higher on average than the spiked amount (Table 1). Regression analysis of the standard curves constructed with plasma substituted for the dilution buffer and using a polyclonal antiserum or monoclonal antibody produced calculated vitellogenin values 70% lower and 42% higher than the spiked amounts on average, respectively (Table 1). Since plasma samples contain plasma, and normally prepared standards do not, it is likely that vitellogenin values are being substantially overestimated. The differences are reflective of the hypothesis that different matrices will produce different effects as far as the standard curves are concerned (Figures 1 and 2). Again, as it is not practical to separate vitellogenin from remaining plasma factors prior to assaying, and it is not very practical to add a variable supply of plasma to the standards, which may or may not contain residual vitellogenin, assays should be conducted with the knowledge that the calculated values are not absolute. This likely explains the large variability in calculated vitellogenin levels between different laboratories employing different assays with different antibodies, on similarly exposed fish.

### 4.3. Effects of Dilution

In addition to affinity and matrix effects, dilutions may impact further the accuracy of calculated vitellogenin concentrations. A method of determining the presence of contamination in plasma samples is by preparing several dilutions of the sample. Sample plasma diluted across several dilutions should produce nearly identical values when each result is multiplied by its dilution factor. If the values fall within the range of the standard curve, the final calculated values should coincide. If they fail to coincide, and instead the calculated values diverge by amounts corresponding to the difference between the dilution factors, this would constitute a strong indicator of a contaminating presence binding specifically to the antibody. Matrix effects, especially at low dilutions, are assumed to be occurring when immunological methods are used [41–43]. These effects should be reduced with dilution, as the typical matrix effect is presumed to result from concentrated nonspecific plasma proteins. Dilution of samples is a common remedy for suspected matrix effects [43]. Unfortunately, the individual results of several dilutions of each sample are rarely reported in endocrine disruption studies. Mylchreest et al. [20] described plasma samples diluted ten-fold producing calculated vitellogenin values which diverged three-fold and upon changing the matrix the divergence increased to four-fold. The effect of antibodies specifically recognizing nontarget molecules in a complex matrix that differs greatly between sample and standard is magnified by correcting for the dilution factors.

#### 4.3.1. Practical Considerations for the Aquatic Toxicologist

When considering the use of ELISAs in toxicology studies, a number of considerations should guide methodological decisions. Most laboratory studies involve only a few fish species, in many cases just a single species. In contrast, field studies can encompass numerous and sometimes varying species of fish based on geography, ecology, and seasonal timing. The generation of polyclonal antisera specific for a single species entails a shorter timeline, low cost (<$1,500 in most cases), and fewer logistic hurdles than does production of a monoclonal antibody. Polyclonal antiserum can be produced and be available for use in 8 weeks where monoclonal antibodies can require several months. In addition, monoclonal antibodies require a cell culture facility and a complicated supply list of chemicals. While it is advisable for laboratory and field studies alike to use species-specific antibodies and purified vitellogenin, field studies may achieve adequate data quality through the use of a polyclonal, species-specific antibody, which can be produced during the planning phase of a study. In contrast, laboratory studies in dedicated aquatic toxicology laboratories may be well advised to invest the time and resources to develop monoclonal species-specific antibodies for the assessment of plasma vitellogenin in exposure experiments. The use of standards prepared in conspecific plasma may remain an unattainable goal for all but the most stringent test standards. 

## 5. Conclusions

ELISAs are a powerful tool in the arsenal of aquatic toxicologists. However, like most other tools, data obtained via the use of ELISAs need to be placed into the context and limitations of the technique. This study focused on assay-specific sources of variability attributable to antibody preparations and sample matrices. However, user-induced sources of variability remain as potential reasons for divergent data points and need to be continually evaluated. Polyclonal antisera are more prone to inflate the quantity of the measured compounds and natural matrices may further alter measured quantities. As a consequence, it is advisable to aquatic toxicologists to avoid comparing absolute calculated plasma vitellogenin concentrations among studies and instead compare either percentages or ratios derived from within the studies to be compared. For example, plasma vitellogenin concentrations in a study can be expressed as % concentration over baseline, as is already widely done when comparing gene mRNA induction [44] or may use a ratio of male vitellogenin in comparison to mean control female plasma vitellogenin, with control female fish assumed to have reproductively optimized plasma vitellogenin concentrations. The latter approach is particularly useful in field studies to compare effects across multiple species (e.g., [45]). With the use of ELISAs in aquatic toxicology likely to increase in the future and with vitellogenin likely to remain an important biomarker for the exposure of oviparous vertebrates to endocrine active compounds, aquatic toxicologists and regulators alike need to educate themselves about the potential and pitfalls of this technique to avoid type 1, false positive, errors.

## Figures and Tables

**Table 1:** The effects of dilution medium on measured concentrations of vitellogenin (mean ± standard error, (sample size)). Plasma from three fathead minnows was analyzed via competitive ELISA using either polyclonal or monoclonal antibody and quantified in one of three separate standard curves prepared in different matrices. Normal method: prepared in phosphate-based dilution buffer. Diluted plasma: unexposed plasma substituted for dilution buffer, where the plasma was diluted with the standards across a range of 1 : 16 to 1 : 1024. Constant plasma: unexposed plasma substituted for dilution buffer with standards added, where the plasma was diluted 1 : 500. Variability in number of samples analyzed reflects sample values outside the linear range of standard curve.

Sample	Polyclonal antiserum			Monoclonal antibody		
	Normal method	Diluted plasma	Constant plasma	Normal method	Diluted plasma	Constant plasma
A	2926 (1)	89 ± 25.4 (3)	94 ± 2.4 (2)	920 ± 49.3 (2)	349 ± 102.6 (3)	547 ± 74.8 (2)
B	1547 (1)	79 ± 16.7 (3)	100 ± 37.7 (2)	1147 (1)	408 ± 147.0 (3)	807 ± 72.7 (2)
C	1997 (1)	77 ± 15.3 (3)	94 ± 39.7 (2)	453 ± 75.2 (2)	206 ± 38.2 (3)	252 ± 54.1 (2)

**Table 2:** Recovery of purified vitellogenin added to dilution buffer and measured via competitive polyclonal ELISA. Multiple dilutions of purified vitellogenin were prepared in PBS/1% BSA and incubated with antivitellogenin rabbit polyclonal antiserum prior to exposure to microtiter plates coated with the identical purified vitellogenin. Recovery is based on purified vitellogenin concentration of 600 *μ*g/mL.

Recovery effort (date)	Repeated vitellogenin analysis	Vitellogenin concentration	Percent of recovery
		mean ± stand. err.	
3/21/2010	642 *μ*g/mL	703.4 ± 32.3 *μ*g/mL	117%
	618 *μ*g/mL		
	788 *μ*g/mL		
	753 *μ*g/mL		
	716 *μ*g/mL		

7/9/2010	492 *μ*g/mL	515 ± 7.2 *μ*g/mL	86%
	520 *μ*g/mL		
	538 *μ*g/mL		
	530 *μ*g/mL		
	510 *μ*g/mL		
	500 *μ*g/mL		

3/11/2011	498 *μ*g/mL	463.2 ± 9 *μ*g/mL	77%
	421 *μ*g/mL		
	436 *μ*g/mL		
	504 *μ*g/mL		
	450 *μ*g/mL		
	476 *μ*g/mL		
	467 *μ*g/mL		
	453 *μ*g/mL		
	462 *μ*g/mL		
